# Clinical and Epidemiological Landscape of Antimicrobial Resistance and Virulence in *Streptococcus* Species

**DOI:** 10.3390/antibiotics15080751

**Published:** 2026-08-03

**Authors:** Telma de Sousa, Catarina Silva, José Eduardo Pereira, Gilberto Igrejas, Patricia Poeta

**Affiliations:** 1 MicroART-Antibiotic Resistance Team, Department of Veterinary Sciences, University of Trás-os-Montes and Alto Douro, 5000-801 Vila Real, Portugal; telmaslsousa@hotmail.com (T.d.S.); xusilva2002@gmail.com (C.S.); 2 Functional Genomics and Proteomics Unit, University of Trás-os-Montes and Alto Douro, 5000-801 Vila Real, Portugal; gigrejas@utad.pt; 3 Associated Laboratory for Green Chemistry, University NOVA of Lisbon, 1099-085 Caparica, Portugal; 4 Department of Veterinary Sciences, University of Trás-os-Montes and Alto Douro, 5000-801 Vila Real, Portugal; jeduardo@utad.pt; 5 CECAV-Veterinary and Animal Research Centre, University of Trás-os-Montes and Alto Douro, 5000-801 Vila Real, Portugal; 6 Department of Genetics and Biotechnology, University of Trás-os-Montes and Alto Douro, 5000-801 Vila Real, Portugal; 7 Veterinary and Animal Research Centre, Associate Laboratory for Animal and Veterinary Science (AL4AnimalS), University of Trás-os-Montes and Alto Douro, 5000-801 Vila Real, Portugal

**Keywords:** *Streptococcus* spp., antibiotic resistance, epidemiology, One Health

## Abstract

Species of the genus *Streptococcus* constitute important pathogens in human and veterinary medicine, being responsible for a wide spectrum of infections ranging from mild illnesses to severe invasive pathologies. Although β-lactams continue to be effective against most species, the global increase in resistance to macrolides, lincosamides, tetracyclines, and, in some cases, reduced susceptibility to penicillin represents a growing challenge for antimicrobial therapy. This review synthesizes the clinical and epidemiological landscape of antimicrobial resistance in the main *Streptococcus* species, including *Streptococcus pyogenes*, *Streptococcus agalactiae*, *Streptococcus pneumoniae*, *Streptococcus dysgalactiae*, and other species of clinical and veterinary relevance, addressing their epidemiological profiles, molecular mechanisms of resistance, and virulence factors. The main genetic determinants involved in resistance are discussed, namely the *erm*, *mef*, and *tet* genes, as well as the impact of alterations in penicillin-binding proteins, horizontal gene transfer, and biofilm formation on the persistence of infections and decreased therapeutic efficacy. Simultaneously, the main virulence factors are analyzed, including polysaccharide capsules, adhesins, toxins, extracellular enzymes, and immune response evasion mechanisms that contribute to the colonization, dissemination, and severity of infections. The importance of epidemiological and genomic surveillance, particularly through whole-genome sequencing, in monitoring the spread of resistant clones and identifying determinants of resistance and virulence is also highlighted. Taken together, the data highlight the need to strengthen programs for the rational use of antimicrobials, to promote integrated surveillance strategies from a One Health perspective, and to deepen knowledge about the interaction between antimicrobial resistance and virulence, in order to improve strategies for the prevention, diagnosis, and treatment of infections caused by *Streptococcus* spp.

## 1. Introduction

*Streptococci* are a genus of Gram-positive bacteria of unique importance in clinical practice, responsible for a wide range of diseases ranging from mild infections, such as pharyngitis and impetigo, to severe invasive conditions, such as sepsis, streptococcal toxic shock syndrome, and rheumatic fever [1]. Species such as *Streptococcus pyogenes* (group A) and *Streptococcus agalactiae* (group B) are among the leading human pathogens, while *Streptococcus pneumoniae* remains one of the most common causes of community-acquired pneumonia, meningitis, and otitis media, particularly in children, the elderly, and immunocompromised individuals [2]. This clinical importance is compounded by the growing spread of multidrug-resistant strains. Although *streptococci* have historically remained susceptible to penicillin, the emergence of resistance to macrolides, lincosamides, and tetracyclines, mediated by genes such as *erm* and *mef*, is alarming, especially in skin and soft tissue infections [3]. Furthermore, reduced tolerance to beta-lactams and the emergence of *S. pneumoniae* strains with intermediate penicillin resistance due to mutations in penicillin-binding proteins (PBPs) have limited therapeutic options [4]. Given this situation, the implementation of high-resolution epidemiological surveillance strategies is imperative. Genomic surveillance, through whole-genome sequencing (WGS), has emerged as an indispensable tool, enabling not only the accurate detection of resistance determinants but also the tracking of emerging clonal lineages, the elucidation of transmission routes, and the prediction of phenotypic profiles.

## 2. Epidemiological Landscape and Pathogenic Profiles of Clinical *Streptococcus* Groups

The genus *Streptococcus* encompasses a highly diverse group of Gram-positive bacteria, typically coccobacillary in shape and arranged in chains. Currently, more than 60 species have been described, which can act as commensals, pathogens, or opportunistic pathogens, affecting both humans and animals (zoonoses) [5,6,7,8,9]. The traditional classification of *streptococci* is based on two fundamental, complementary criteria: the pattern of hemolysis observed on blood agar medium [10] and the antigenic composition of the cell wall, according to the Rebecca Lancefield classification [11] (Table 1). Regarding hemolysis, three phenotypes are distinguished, reflecting their capacity to destroy or modify erythrocytes. In β-hemolysis, there is production of hemolysins (streptolysins S and O) that completely lyse erythrocytes; most β-hemolytic species are associated with aggressive and invasive infections [12]. However, it is important to emphasize that, although streptolysins S and O are determinants of beta-hemolysis in many *streptococci*, this mechanism is not universal [13,14]. In *Streptococcus agalactiae*, for example, hemolysis is due to the production of a beta-carotenoid pigment called garnetane, whose presence is so characteristic that it allows the presumptive identification of this species in specific culture media, such as Granuloma agar. Furthermore, streptolysin O is oxygen-labile, being inactivated under aerobic conditions; its hemolytic activity can therefore be optimally visualized by stab inoculation into blood agar, where a distinct zone of hemolysis develops along the anaerobic track of the inoculation [14]. In contrast, streptolysin S is oxygen-stable and accounts for the typical beta-hemolytic halo observed around surface colonies grown under aerobic conditions. Beyond their differential oxygen sensitivity, these toxins present important, distinct biochemical and clinical features [13]. Streptolysin O is a thiol-activated, pore-forming cytolysin that binds to membrane cholesterol. It is highly antigenic, eliciting anti-streptolysin O antibodies, the titers of which are widely used as serological markers for the diagnosis of streptococcal infections. Streptolysin S, on the other hand, is a small, non-immunogenic cytotoxin that exerts potent damaging effects on leukocytes, platelets, and other mammalian cell lines. Notably, streptolysin S is not restricted to *Streptococcus pyogenes*, as it is also produced by groups C and G *streptococci*, as well as by the zoonotic pathogen *Streptococcus iniae* [13,14,15]. In α-hemolysis, *streptococci* produce hydrogen peroxide (H_2_O_2_), which oxidizes the heme of red blood cells without complete lysis. In γ-hemolysis, *streptococci* do not produce significant hemolysins under standard culture conditions; however, the absence of hemolysis does not imply a lack of virulence [12]. *Streptococcus gallolyticus*, for example, is strongly associated with endocarditis and colorectal neoplasia, an association supported by multiple cohort studies and systematic reviews that recommend screening for colonic lesions after isolation of this microorganism [16].

Rebecca Lancefield, in the 1930s, demonstrated that β-hemolytic and some non-hemolytic *streptococci* possess specific polysaccharide antigens in the cell wall (groups A to V) [11]. This classification remains useful in routine practice, although not all species fit perfectly. Group A *Streptococcus* (GAS), represented exclusively by *Streptococcus pyogenes*, is β-hemolytic, bacitracin-sensitive, and has a strictly human reservoir [17]. It is responsible for suppurative infections such as pharyngitis (with asymptomatic carriers in 5-20% of the population), impetigo, erysipelas, necrotizing fasciitis, and streptococcal toxic shock syndrome, as well as non-suppurative sequelae mediated by cross-immunity (acute rheumatic fever and post-streptococcal glomerulonephritis) [18,19]. For laboratory diagnosis, oropharyngeal secretion or lesion sampling is performed, followed by culture on blood agar (β-hemolysis, bacitracin-sensitive, PYR-positive) and rapid antigen tests/ASLO (anti-streptolysin O) in sequelar infections. Treatment is generally managed with penicillin V or amoxicillin (first-line, as *S. pyogenes* is almost universally sensitive) [20]. In allergic patients, erythromycin/azithromycin or clindamycin are used (although resistance to macrolides is increasing). In severe cases, clindamycin is added to inhibit toxin production. Robust clinical evidence, namely randomized trials and Infectious Diseases Society of America guidelines, confirms the efficacy of penicillin and the utility of clindamycin in toxic forms [21].

Group B, whose main agent is *Streptococcus agalactiae*, is also β-hemolytic (although non-hemolytic variants exist) and is distinguished by a positive CAMP test [22]. It is a commensal inhabitant of the human gastrointestinal and genitourinary tracts (about 18–35% of pregnant women are colonized) but causes severe invasive disease in newborns (sepsis, pneumonia, and meningitis) via vertical transmission, as well as in pregnant or immunocompromised adults, where it causes urinary tract infections and chorioamnionitis [22,23,24]. In non-pregnant adults, the epidemiology and clinical manifestations of *Streptococcus agalactiae* take on a distinctly different character. In this population, frequently affecting men, the elderly, and those with chronic illnesses, the infection acts opportunistically. Severe disease is almost exclusive to individuals with underlying medical conditions that compromise the immune system, such as diabetes mellitus, obesity, cancer, kidney disease, or liver disease [25,26]. It also has veterinary importance as an agent of bovine mastitis and can be transmitted by fish or camels zoonotically. For treatment, penicillin G (or ampicillin) is used for intrapartum prophylaxis or clinical infections. In allergic patients, cefazolin or erythromycin/clindamycin are used (with prior sensitivity culture) [27,28,29].

Groups C and G encompass several β-hemolytic species that react with C or G antigens and sometimes both. Notable species include *Streptococcus dysgalactiae* subsp. *equisimilis* (SDSE), which causes pharyngitis (clinically indistinguishable from group A), cellulitis, bacteremia, and endocarditis in humans [30], and *Streptococcus canis*, which is associated with infections in dogs and cats and has zoonotic potential; contact with domestic animals can transmit resistant strains [31]. *Streptococcus equi* subsp. *zooepidemicus* is an equine and bovine pathogen, occasionally transmitted by contaminated dairy products, causing nephritis and toxic shock syndrome [32,33]. Diagnosis is based on β-hemolysis, Lancefield group C/G (via serum tests), and biochemical tests: SDSE is optochin-resistant and PYR-negative (unlike GAS). Treatment is with penicillin G (SDSE and *Streptococcus equi* subsp. *zooepidemicus* are sensitive); for allergic patients, cephalosporins or macrolides are used if susceptibility is present. *S. canis* is generally sensitive to penicillin, but resistance in swine should be monitored [34].

In the non-enterococcal group D, the most relevant species is *S. gallolyticus* (formerly *S. bovis*), which is γ-hemolytic, grows in bile-esculin but does not share the characteristics of enterococci [35]. This microorganism is known for its strong association with infectious endocarditis and colon neoplasia, sometimes serving as a marker for occult colorectal lesions. A recent meta-analysis showed that about 30% of patients with *S. gallolyticus* bacteremia present with colonic adenomas or carcinomas, justifying systematic colonoscopy [36,37]. Diagnosis is made by isolation in blood culture (not optochin-sensitive), growth in bile and positive esculin (Lancefield D), and identification by MALDI-TOF or 16S sequencing [38,39]. Treatment of endocarditis requires penicillin G or ceftriaxone for ≧4 weeks (vancomycin in allergic patients). In isolated bacteremia, ampicillin is used. A combination with gentamicin may be used in severe endocarditis [37].

Group R/S is represented by *Streptococcus suis*, a zoonotic pathogen of great importance. Depending on the strain, it can be β-hemolytic or non-hemolytic, and its polysaccharide capsule defines more than 30 serotypes, with types 2 and 14 being the most frequently involved in human disease. Infection occurs mainly in individuals exposed to swine or raw pork [40,41]. Diseases in humans include mainly acute meningitis (43–50% of cases), sepsis, pneumonia, and ophthalmitis (or endophthalmitis). Typical manifestations are fever, headache, and neck stiffness, with a high incidence of post-infection deafness (about 53%). Fatal cases are rare (2.9% in this group) but can occur in septic shock. Joint involvement and endocarditis have been reported (6–16%) [42,43]. In swine, it causes polyserositis (arthritis, meningitis, and septic pneumonia) in piglets and sows, with high mortality in herds, a multisystemic disease termed “porcine *streptococcosis*.” Virulence is associated with the capsule (anti-phagocytic), suilysin (pneumolysin-type toxin), surface proteases, and muramidases; virulence genes are horizontally transferred by plasmids and bacteriophages [44,45]. Diagnosis is made by blood or CSF culture (β-hemolysis, bacitracin-resistant), and identification by MALDI-TOF or real-time PCR can detect *S. suis* directly [46]. Treatment uses penicillin G (robust doses) or ceftriaxone for meningitis (vancomycin is used in allergic patients). Adjuvant dexamethasone reduces neurological complications (following the protocol for bacterial meningitis), and attention to septic shock is necessary [47,48].

Beyond the groups with known Lancefield antigens, there is a large set of non-typeable *streptococci*, which do not react with available sera. These include *Streptococcus pneumoniae* and the *viridans* group *streptococci* (VGS). *S. pneumoniae* is α-hemolytic, optochin-sensitive, and bile-soluble and constitutes the main agent of community-acquired pneumonia, bacterial meningitis, and acute otitis media [49,50,51]. The reservoir is the human nasopharynx (5–70% carriers, especially children), with transmission by respiratory droplets and a peak in winter/spring. Diseases include community-acquired pneumonia, bacterial meningitis, acute otitis media, sinusitis, and bacteremia/septicemia, especially in the elderly and children. In adults, it can cause exacerbations of chronic disease, septic arthritis, and bacterial peritonitis [50,52]. Diagnosis is based on Gram stain (diplococci in culture), blood agar culture (α-hemolysis, convex “draftsman” or “coin-shaped” colonies), optochin sensitivity, bile solubility, detection of C-polysaccharide in fluid (pneumonia), and PCR or rapid urinary antigen tests. Treatment is with penicillin G (or amoxicillin) for mild infections, ceftriaxone/cefotaxime for severe cases (meningitis/severe pneumonia), and vancomycin if resistance is documented [51]. The VGS group is a heterogeneous set of most α-hemolytic or γ-hemolytic species. Based on phylogenetic and phenotypic characteristics, *viridans* are currently divided into six major groups: the *mitis*, *sanguinis*, *anginosus*, *mutans*, *salivarius*, and *bovis* groups [53,54,55]. Members of the *mitis* group, including *S. mitis* and *S. oralis*-related species, are among the most abundant oral colonizers and are frequently associated with infective endocarditis and bloodstream infections, particularly in immunocompromised patients [56,57]. The *sanguinis* group (*S. sanguinis* and *S. gordonii*) plays an important role in early dental biofilm formation and is recognized as one of the leading causes of native valve infective endocarditis due to its strong ability to adhere to damaged cardiac endothelium [58,59]. In contrast, the *mutans* group, represented mainly by *S. mutans* and *S. sobrinus*, is considered the principal etiological agent of dental caries because of its remarkable capacity to metabolize dietary carbohydrates into organic acids and synthesize extracellular polysaccharides that promote persistent biofilm formation [60]. The *anginosus* group (*S. anginosus*, *S. constellatus*, and *S. intermedius*) is distinguished by its marked propensity to cause deep-seated abscesses involving the brain, liver, lungs, and intra-abdominal tissues [61]. The *S. anginosus* group tends to form deep abscesses in the liver, brain, and other locations [62]. In veterinary medicine, non-typeable *streptococci* such as *S. uberis* and *S. parauberis* are predominant causes of bovine mastitis, while *S. iniae* is a fish pathogen that can cause cellulitis in humans after handling aquaculture products [63,64]. Recent epidemiological studies reinforce the zoonotic and occupational role of these species, requiring a “One Health” approach in their control. In developed countries, VGS remain among the three most common etiological agents of community-acquired infective endocarditis, accounting for approximately 20–30% of native valve cases, although their relative incidence has declined with the increasing prevalence of healthcare-associated *Staphylococcus aureus* infections [65,66]. The *viridans* group grows on blood agar with mild α-hemolysis, is optochin-resistant, and its identification is confirmed by biochemical tests or MALDI-TOF [54]. The treatment of *viridans* endocarditis is performed with penicillin G + gentamicin (usually with a low MIC). In dental caries, treatment is mechanical (biofilm removal, fluoride) without routine antibiotics; abscesses from the *anginosus* group require drainage plus penicillin or ceftriaxone [67,68]. There is high tolerance to various drugs due to biofilm formation [68].

## 3. Antimicrobial Resistance Patterns in *Streptococci*

Antimicrobial resistance (AMR) in *streptococci* has been increasing globally over the past few decades, driven by multiple molecular mechanisms and clinical practices. The intrinsic and acquired resistance mechanisms in *streptococci* (including mutations, horizontal gene transfer, efflux pumps, target modifications, and biofilm formation) are varied [34], as are the resistance patterns across the various streptococcal species: *S. pneumoniae*, *S. pyogenes*, *S. agalactiae*, *viridans* group *streptococci*, and *S. dysgalactiae*. Recent global and European surveillance data (from the past 10 years) highlight rising resistance rates, marked regional differences (e.g., higher AMR in southern Europe), and determining factors (antibiotic use and vaccination) [69,70].

*Streptococci* exhibit a variety of AMR mechanisms, including target alteration (such as mutations in *pbp* genes encoding penicillin-binding proteins, resulting in low-affinity PBPs in *Streptococcus pneumoniae* and *viridans* group *streptococci*) [71], modification of the ribosomal target (e.g., methylation of 23S rRNA by Erm methyltransferases [genes *erm(B)* or *erm(TR)*] [72], conferring the *MLS_B_* phenotype of resistance to macrolide-lincosamide-emepenotrin) [73], efflux pumps (such as the *mef* gene in *S. pyogenes*), and the ability to form biofilms that hinder antibiotic penetration. In *streptococci* in general, resistance to aminoglycosides is intrinsic at low concentrations due to poor drug uptake, which is why synergy with β-lactams is required to achieve a bactericidal effect [24]. Additionally, aminoglycoside-inactivating genes (AAC, APH, ANT) can confer high levels of resistance, although they are rare in *S. pyogenes* [72,74]. Although direct enzymatic inactivation (β-lactamases) is rare in this genus [75,76], horizontal gene transfer mediated by natural transformation (in *S. pneumoniae* and *viridans*) [75,76] or by conjugative elements [such as Tn916 transposons carrying *tet*(M)] is a major driver of resistance spread [77]. These mechanisms manifest differently depending on the species, shaping specific epidemiological profiles and therapeutic challenges. The reported antimicrobial resistance rates among the most clinically relevant *Streptococcus* species are summarized in Table 2, highlighting species-specific differences in resistance to commonly used antimicrobial classes.

In *Streptococcus pneumoniae* (pneumococci), the resistance to β-lactams is predominantly due to low-affinity PBPs (target alteration), often associated with multidrug-resistant clones [78,79]. As for aminoglycosides, low intrinsic uptake necessitates a synergistic combination with β-lactams for bactericidal effect; inactivating genes are rare. Data from the ECDC (EU/EEA, 2024) show that 17.3% of invasive isolates are “non-wild-type” for penicillin and 19.0% are resistant to macrolides (mainly due to rRNA methylation via *erm* or efflux via *mef*), with a marked north-south gradient in Europe (higher rates in the south) [80]. The use of pneumococcal conjugate vaccines (PCVs) has reduced the circulation of resistant serotypes. Clinically, resistance to penicillins and cephalosporins may require higher doses or second-line antibiotics, while high resistance to macrolides limits their empirical use in community-acquired pneumonia [78,79,81].

*Streptococcus pyogenes* (GAS—group A), on the other hand, remains universally susceptible to penicillin, although point mutations in *pbp2x* have been described experimentally without any apparent impact [82,83]. Resistance to macrolides/lincosamides is mediated by *erm* genes (constitutive or inducible *MLS_B_* phenotype) or *mef* (efflux), with rates ranging from 15 to 30% in- Europe [84]. Resistance to tetracyclines [e.g., via *tet(M)* or *tet(O)*] is common (approximately 20–30%) [85], while vancomycin and linezolid remain fully effective. For patients allergic to penicillin, high rates of resistance to macrolides and clindamycin necessitate susceptibility testing before using these agents. The diagnosis should include the D-zone test to detect inducible resistance to clindamycin, thereby avoiding false-positive results [86].

For *Streptococcus agalactiae* (group B), β-lactams remain the first-line treatment, with very low resistance rates (approximately 1.7% globally for penicillin and 3.1% for ampicillin) [87]. In contrast, resistance to tetracyclines is highly prevalent, affecting approximately 80% of human isolates globally. This widespread resistance is largely attributed to the historical clonal expansion of human-adapted GBS lineages carrying the *tet(M)* gene, rather than to contemporary tetracycline use [88,89]. Resistance to macrolides and lincosamides has also increased substantially in recent decades, with erythromycin resistance commonly exceeding 30–40% and clindamycin resistance ranging from 25 to 35%, primarily- due to the dissemination of *erm* genes and, to a lesser extent, *mef*-mediated efflux mechanisms [90]. Fluoroquinolones have also shown increased resistance (levofloxacin close to 50% in some series) [91]. Current evidence suggests that these resistance trends are more closely associated with the widespread use of macrolides in the general population than with intrapartum antibiotic prophylaxis, as similar resistance rates have been reported in countries where universal GBS prophylaxis is not routinely implemented [22,23]. Consequently, penicillin remains the drug of choice for intrapartum prophylaxis. In patients with severe β-lactam allergy, clindamycin is recommended as the preferred second-line agent only when antimicrobial susceptibility testing confirms susceptibility, whereas vancomycin should be used when resistance to clindamycin is detected or susceptibility results are unavailable [92,93].

*Viridans* group *streptococci* (VGS), a heterogeneous group of oral species, exhibit moderate intrinsic resistance to aminoglycosides and variations in PBPs that confer intermediate resistance to penicillins (in some subspecies, up to 10–15%) [94]. Resistance rates to macrolides and clindamycin are high (20–50%, mediated by *erm/mef*), while resistance to tetracyclines reaches 60–80% [mediated by *tet(M)*] [95,96]. All remain susceptible to vancomycin and linezolid. Systematic epidemiological data are scarce due to species diversity, but in EVG endocarditis, standard treatment with high-dose penicillin combined with gentamicin assumes susceptibility; treatment failure may occur in the presence of biofilms on valvular vegetations [97]. Fluoroquinolone resistance has been associated with mutations in the quinolone resistance-determining regions (QRDRs) of *gyrA* and *parC*, whereas biofilm-associated tolerance substantially reduces the activity of multiple antimicrobial classes despite the absence of conventional resistance determinants [98,99]. The oral cavity functions as an important reservoir of mobile genetic elements, facilitating horizontal transfer of resistance genes between commensal *streptococci* and other clinically relevant pathogens. Finally, *Streptococcus dysgalactiae* (groups C/G) has a profile like that of GAS, universal susceptibility to β-lactams [100], moderate resistance to macrolides (10–20%, *MLS_B_* phenotype) and tetracyclines, and full susceptibility to glycopeptides and linezolid [101].

Globally, surveillance data (WHO, ECDC, and GLASS) show an increase in antimicrobial resistance in *Streptococcus* over the past decade, with significant geographical variations. For pneumococci, the proportion of invasive isolates resistant to at least one antimicrobial has been rising, with a doubling of the incidence of resistance to macrolides and non-wild-type penicillin in Europe between 2020 and 2024. For GAS, post-pandemic outbreaks of COVID-19 in 2022–2023 reinforced the importance of penicillin as a first-line treatment, but resistance rates to erythromycin in Europe range from 20% to 30%. In GAS, meta-analyses indicate an overall average resistance of 35% to erythromycin and 29% to clindamycin, with resistance to tetracyclines reaching 80%. Regions with high macrolide consumption (southern Europe and East Asia) have the highest resistance rates, while pneumococcal vaccination has successfully reduced resistant serotypes [84,102,103].

The clinical and public health implications are direct, as resistance to macrolides limits their empirical use in pneumococcal pneumonia and in prophylaxis and treatment of penicillin-allergic patients for GBS and GAS. Treatment failures due to beta-lactam resistance in pneumococcal infections require antibiotic escalation or switching. Streptococcal biofilms in endocarditis and implant-associated infections require prolonged combination therapies. Antimicrobial stewardship programs are essential, encompassing the rational use of antibiotics and vaccination, as well as standardized continuous surveillance. Public health relies on networks such as EARS-Net and GLASS to guide policy. Regarding laboratory methods, AMR detection faces challenges such as the need to confirm penicillin MICs via E-test or microdilution, given that the oxacillin disk is only a screening tool. Another challenge is the D-zone test for inducible clindamycin resistance in GAS and GBS, and the inability of routine methods to detect biofilm-associated tolerance. Standardization between EUCAST and CLSI and the growing use of molecular methods such as PCR and WGS for surveillance complement the phenotype but do not replace it for direct clinical decision-making.

## 4. Virulence Factors and Their Interplay with Antimicrobial Resistance

The interaction between pathogenic microorganisms and their host is defined by a continuous and complex evolutionary arms race. For a pathogen to successfully establish an infection, it is imperative that it overcome the robust physical barriers and sophisticated defenses of the host’s immune system [104,105,106]. It is in this context of survival and adaptation that virulence mechanisms come into play, a diverse set of genetic, biochemical, and structural strategies that give the microorganism the ability to colonize, invade tissues, evade the immune response, and ultimately cause cellular damage [104,105,106,107,108].

*Streptococcus* spp. has a pathogenic potential largely determined by the repertoire of virulence factors encoded by different species. Despite these species sharing common structural characteristics as Gram-positive cocci, they exhibit diversity in their ability to colonize host tissues, evade immune defenses, acquire nutrients, and cause disease. Consequently, the clinical manifestations associated with different *streptococci* species range from asymptomatic colonization to severe invasive infections, reflecting species-specific adaptations to distinct ecological niches and host environments [105].

**Figure 1:**
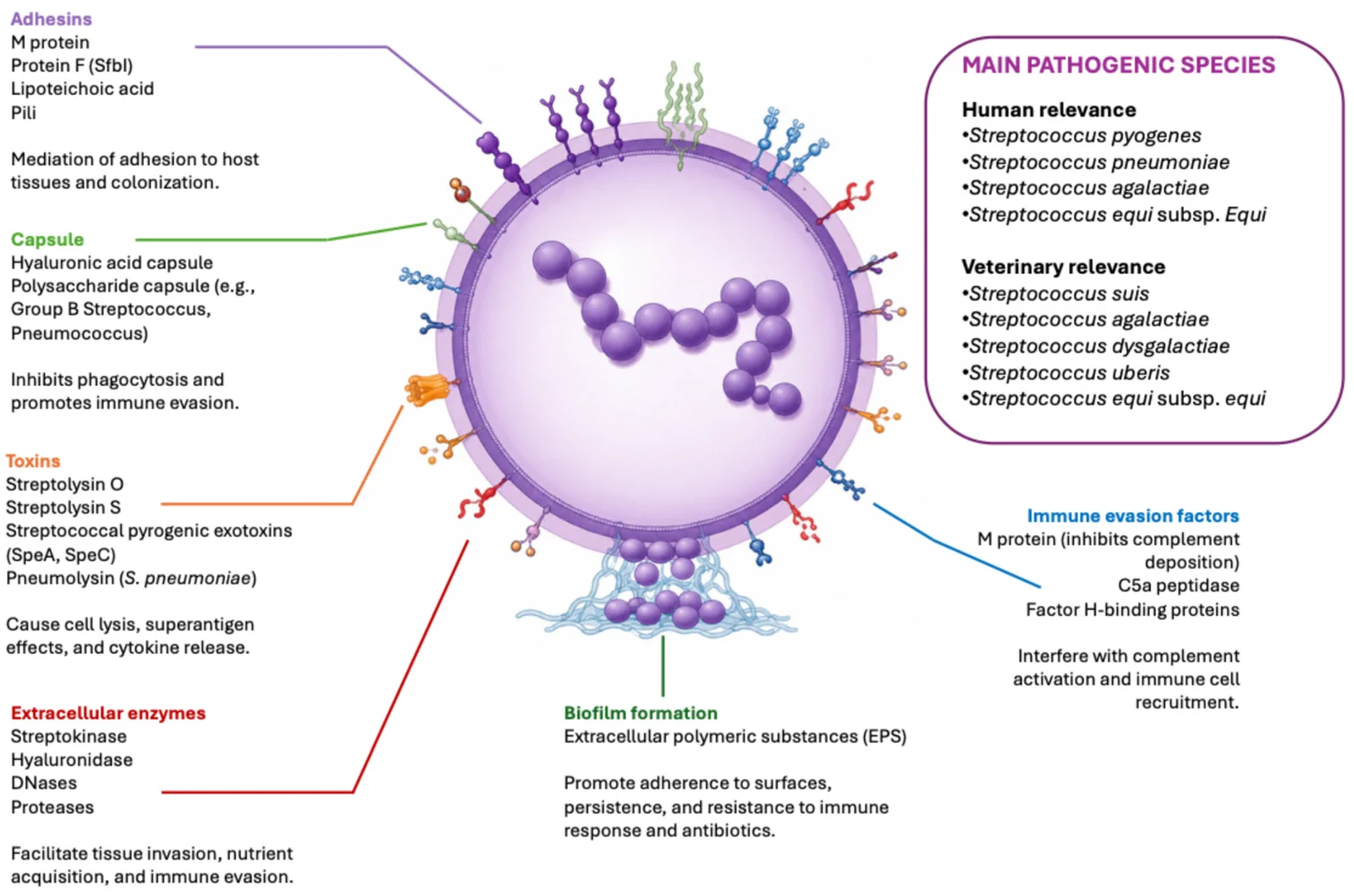
Virulence factors and infection mechanisms. Representation of surface structures and secreted molecules that facilitate colonization, tissue invasion, and evasion of the host’s immune system by the main pathogenic *Streptococcus* spp. species impacting humans and veterinary health.

Among the numerous species of the genus, *Streptococcus pyogenes*, *Streptococcus pneumoniae*, *Streptococcus agalactiae*, and *Streptococcus equi* subsp. *equi* are responsible for the greatest disease burden in humans, while the species of greatest veterinary relevance include *Streptococcus suis*, *Streptococcus agalactiae*, *Streptococcus dysgalactiae*, *Streptococcus uberis*, and *Streptococcus equi* subsp. *equi*, associated with significant diseases in swine, cattle, sheep, and horses [44,109,110,111,112,113,114]. These pathogens possess a wide range of virulence determinants, including polysaccharide capsules, adhesins, cytolysins, immune evasion proteins, extracellular enzymes, and biofilm-forming capabilities [115,116] (Figure 1). Although some virulence mechanisms are conserved across various species, such as biofilm formation and adhesins, others are unique and responsible for the distinct pathogenic profiles, tissue tropism, and disease severity observed among *streptococci*, such as the cytotoxin pneumolysin produced by *Streptococcus pneumoniae* [117].

### 4.1. Streptococcus pyogenes

Colonization by *S. pyogenes* occurs predominantly in the oropharynx and on the skin, representing the starting point for a vast spectrum of infections ranging from acute pharyngitis to severe invasive forms such as necrotizing fasciitis and streptococcal toxic shock syndrome [118]. The process initiates with adhesion to the host epithelium, mediated by a set of highly adaptive surface structures. It is crucial to note that while the M protein is a hallmark and primary virulence factor of *S. pyogenes*, its role is not prominent or well-documented in other major streptococcal pathogens, such as *S. pneumoniae*, which rely on different primary virulence mechanisms [119,120]. The M protein plays a central role in *S. pyogenes* pathogenesis, promoting binding to extracellular matrix components such as fibrinogen and fibronectin, while simultaneously interfering with host immune mechanisms, allowing for stable attachment and the initiation of colonization. Given the high diversity of this protein, more than 200 emm types have been identified. Epidemiologically, invasive diseases worldwide are frequently driven by a specific subset of emm types, most notably emm1, emm3, emm12, emm28, and emm89 [121,122,123]. Among these, the emm1 type is historically and consistently associated with the highest risk of severe invasive infections and streptococcal toxic shock syndrome. In parallel, protein F (SfbI) reinforces adhesion to fibronectin and facilitates internalization into epithelial cells, contributing to the persistence and recurrence of infections [124,125,126]. Lipoteichoic acid acts during the initial contact phases, promoting hydrophobic interactions with the cell surface and stabilizing bacterial adhesion [126].

Once colonization is established, *S. pyogenes* reveals a remarkable capacity for immune system evasion, which is essential for its high virulence and invasive potential [127]. The M protein continues to play a determinant role by blocking complement-mediated opsonization and hindering phagocytosis by neutrophils through the inhibition of C3b deposition on the bacterial surface [128,129,130]. The hyaluronic acid capsule further contributes to molecular mimicry, as it structurally resembles human tissues, thereby reducing immunological recognition. In parallel, the production of C5a peptidase degrades the chemotactic factor C5a, significantly decreasing the recruitment of inflammatory cells to the infection site and facilitating increased bacterial survival within the tissues [131,132].

As the infection progresses, the tissue dissemination capacity of *S. pyogenes* is amplified by the production of a broad array of extracellular toxins and enzymes. Streptolysins O and S promote direct cell lysis, affecting erythrocytes, leukocytes, and platelets, which contributes to extensive tissue damage and immune system evasion [133,134]. In parallel, streptococcal pyrogenic exotoxins (SpeA, SpeC, among others) act as superantigens, inducing massive T-lymphocyte activation and the uncontrolled release of cytokines, a central mechanism in streptococcal toxic shock syndrome [135,136]. Enzymes such as streptokinase facilitate the degradation of fibrin clots, promoting dissemination throughout the tissues, while hyaluronidase degrades the extracellular matrix, clearing the path for deep invasion and necrosis [134,137]. Streptokinase is a secreted plasminogen activator that forms a high-affinity complex with human plasminogen, inducing a conformational change that generates active plasmin without requiring proteolytic cleavage. The resulting plasmin degrades fibrin clots and extracellular matrix components, facilitating bacterial dissemination through host tissues and enabling escape from sites of localized infection [126,138]. Moreover, plasmin bound to the bacterial surface protects *S. pyogenes* from opsonophagocytic killing and enhances the degradation of complement components and other host defense molecules, thereby promoting immune evasion [139]. Streptokinase also acts synergistically with other extracellular enzymes, including hyaluronidase, DNases, and the cysteine protease SpeB, to promote tissue invasion and bacterial spread during severe infections such as necrotizing fasciitis and streptococcal toxic shock syndrome [126,140]. Genetic diversity within the ska gene has resulted in distinct streptokinase variants with different affinities for human plasminogen, reflecting host adaptation and contributing to differences in strain virulence [141]. Experimental studies have demonstrated that deletion or inactivation of *ska* significantly reduces bacterial dissemination and virulence in animal infection models, confirming streptokinase as a critical determinant of invasive group A streptococcal disease [126,141].

Although classically considered an extracellular pathogen, *S. pyogenes* can also survive temporarily within host cells, including epithelial and phagocytic cells, which contributes to the persistence and recurrence of infections. This intracellular niche provides partial protection against antibiotics and the effector mechanisms of the immune system. Additionally, the ability to form structured bacterial communities and interact with biofilms enhances resistance in chronic infections, particularly in the skin and tonsils, reinforcing the complexity of its pathogenesis [137].

### 4.2. Streptococcus agalactiae

Capsular polysaccharide (CPS) serotyping remains the principal epidemiological marker of *S. agalactiae*, with ten serotypes (Ia, Ib, and II-IX) currently recognized. Their distribution varies according to geographical region and clinical presentation. Serotypes Ia, III, and V account for the majority of maternal colonization and invasive disease in adults worldwide, whereas serotype III, particularly the hypervirulent CC17 lineage, is strongly associated with late-onset neonatal disease and meningitis [142,143,144]. Overall, serotypes Ia, Ib, II, III, and V are responsible for approximately 95–98% of neonatal invasive infections globally, making capsular serotype surveillance essential for monitoring disease epidemiology and informing vaccine development [142,145]. Accordingly, several multivalent conjugate vaccines targeting the most prevalent serotypes are currently in advanced clinical development, with the aim of preventing maternal colonization and neonatal disease [146].

Colonization by *S. agalactiae* constitutes the essential first step for the development of invasive infection, being particularly relevant in pregnant women, neonates, and immunocompromised individuals [147]. This bacterium preferentially establishes itself within the gastrointestinal and genitourinary tracts, where it interacts with the host epithelium through a diverse array of surface adhesins [148]. Proteins such as FbsA and FbsB mediate binding to fibrinogen and facilitate cellular adhesion and invasion, while Lmb and ScpB contribute to laminin binding and the colonization of epithelial surfaces [149,150,151]. Pili reinforce mucosal adhesion and promote biofilm formation, increasing bacterial persistence and facilitating vertical maternal-fetal transmission [148]. This communal state confers greater tolerance to environmental stress and contributes to persistent or recurrent infections, reinforcing the role of *S. agalactiae* as an efficient colonizer and opportunistic pathogen [152].

Once colonization is established, *S. agalactiae* activates multiple immune system evasion mechanisms that facilitate its survival within the host. The sialic acid-rich polysaccharide capsule is one of the primary virulence factors, functioning through a molecular mimicry mechanism that reduces immunological recognition and complement deposition, thereby decreasing opsonization and phagocytosis by neutrophils [153,154]. The surface-associated C5a peptidase ScpB further promotes immune evasion by cleaving the complement-derived chemoattractant C5a, thereby reducing neutrophil recruitment, while also contributing to epithelial adhesion [155,156]. Additionally, the bacterium expresses the CAMP factor, which enhances cell lysis in cooperation with hemolysins from other species, contributing to tissue damage and adaptation to the microbial environment [157,158,159]. Although the CAMP factor is a characteristic feature of *S. agalactiae* and remains widely used for laboratory identification through the CAMP test, its contribution to this bacterium’s virulence is considered limited and is not essential for invasive disease [23,160]. Current evidence indicates that GBS pathogenicity depends primarily on other virulence determinants. Hyaluronate lyase (HylB) facilitates tissue invasion by degrading hyaluronic acid and modulating the host immune response, and in hypervirulent strains, particularly those of the CC17 clonal complex, the expression of the HvgA adhesin is associated with blood-brain barrier invasion and neurotropism, representing a key factor in the pathogenesis of neonatal meningitis [161,162]. The invasive potential of this species is strongly associated with the production of enzymes and toxins that promote tissue dissemination and cellular damage [162]. The β-hemolysin/cytolysin plays a central role in cell membrane injury, contributing to intense inflammation and the disruption of epithelial barriers, which facilitates bacterial translocation to sterile tissues, including the central nervous system [162]. This process is further reinforced by the action of enzymes such as hyaluronidase, which degrades components of the extracellular matrix, and DNases, which promote evasion of neutrophil extracellular traps (NETs) [163]. Additional proteases contribute to the degradation of host proteins, enhancing the capacity for bacterial dissemination.

### 4.3. Streptococcus pneumoniae

Colonization by *S. pneumoniae* occurs primarily in the nasopharynx and constitutes the first step in the development of pneumococcal disease [164,165]. Transmission occurs through respiratory droplets, and asymptomatic colonization is very common, especially in children, who represent the main reservoir of the bacteria. Progression to disease occurs when the bacteria overcome local barriers and reach normally sterile sites, such as the middle ear, lungs, or bloodstream [166,167].

Adhesion to host cells is mediated by various surface structures, including choline-binding proteins (CBPs), such as PspC, which recognize host receptors and promote binding to the respiratory epithelium [168,169]. Other structures, such as pili, also contribute to bacterial attachment, facilitating persistent colonization of the nasopharynx [168,170]. The polysaccharide capsule represents the main virulence factor of *S. pneumoniae*, exerting a strong anti-phagocytic action by inhibiting opsonization by C3b and hindering the action of the complement system and neutrophils [170]. The existence of multiple capsular serotypes contributes to differences in virulence, colonization capacity, and association with antimicrobial resistance, and some serotypes are more frequently associated with severe invasive diseases [170]. The capsule is also the main target of pneumococcal vaccines due to its high immunogenicity [171].

Cell wall components, namely teichoic and lipoteichoic acids, play an important role in the host’s inflammatory response [172]. These elements contain phosphocholine residues that activate the complement system and induce the production of pro-inflammatory cytokines, contributing to the inflammation associated with pneumococcal infection [110,172]. In parallel, choline-binding proteins include several relevant virulence factors, such as LytA, an autolysin that promotes bacterial lysis [118] and the release of other virulence factors, such as PspA (pneumococcal surface protein A), which interferes with complement deposition and reduces phagocytosis [173] and PspC, which acts as an adhesin and facilitates invasion of the respiratory epithelium and even the blood-brain barrier, also contributing to immune system evasion [169,174].

Another important virulence factor is pneumolysin, a cytolytic toxin released after bacterial lysis, which forms pores in the membranes of host cells, causing direct cell damage and cell death [175]. In addition to its cytotoxic effect, pneumolysin activates intense inflammatory responses and interferes with the complement system, contributing significantly to the pathogenesis of invasive infection [117]. LPxTG proteins, anchored to the cell wall by sortases, include several enzymes and structures involved in tissue invasion, such as hyaluronidase, which degrades the extracellular matrix and facilitates bacterial dissemination; neuraminidase [175,176], which removes sialic acid from glycoconjugates and exposes receptors for adhesion; and IgA1 protease, which cleaves immunoglobulin A and facilitates mucosal colonization [177,178]. Pili, also belonging to this group, promote adhesion to the epithelium and induce the production of inflammatory cytokines, contributing to colonization and virulence [170,176].

Additionally, surface lipoproteins play essential roles in bacterial survival, notably in the acquisition of metallic nutrients such as iron and magnesium [168]. Among them, PsaA, involved in magnesium transport [179,180], and PiuA and PiaA proteins, associated with iron acquisition, stand out as fundamental for adaptation to the host environment [181,182]. Pneumococcal infection begins with asymptomatic colonization, which can progress to invasive disease when the bacteria cross mucosal barriers and reach sterile sites, causing pathologies such as pneumonia, otitis media, sinusitis, meningitis, bacteremia, and sepsis [110,183]. Community-acquired pneumonia is the most common clinical manifestation, while invasive forms are more frequent and severe in children, the elderly, and immunocompromised individuals [184,185].

### 4.4. Streptococcus dysgalactiae

The pathogenicity of *S. dysgalactiae* results from the coordinated action of multiple virulence factors that promote colonization, tissue invasion, evasion of the immune response, and persistence in the host [30]. Initial adhesion to host cells is mediated by a set of surface proteins, including fibronectin-binding proteins (FnbA, FnbB, and GfbA), laminin-binding proteins (Lmb), and M-like proteins, which facilitate interaction with components of the extracellular matrix and promote tissue colonization [186,187,188]. Some of these adhesins also promote bacterial internalization in epithelial cells, allowing the bacteria to remain protected from the action of the immune system during the initial stages of infection [189].

Among the main virulence determinants, streptolysin S, encoded by the sag operon, stands out as responsible for cytolytic activity and the destruction of host cells [13]. Additionally, the C5a peptidase (scpA/scpB) reduces neutrophil recruitment to the site of infection by degrading the C5a chemotactic factor, while M-like proteins contribute to immune evasion, hindering opsonization and phagocytosis [132,190]. Some strains also possess genes encoding superantigens, DNases, and other virulence proteins acquired through mobile genetic elements, increasing pathogenic potential and adaptability to different hosts [191].

One of the most relevant aspects of *S. dysgalactiae* biology is its high capacity to form biofilms. These three-dimensional structures consist of bacterial communities embedded in an extracellular matrix composed of polysaccharides, proteins, and extracellular DNA, providing protection against adverse environmental factors, antibiotics, and host defense mechanisms [192]. Biofilm formation is associated with the expression of several genes, including *brpA*-like, *fbpA*, *htrA*, and *sagA*, whose regulation varies between planktonic and sessile cells [186]. Studies conducted on abiotic surfaces, human keratinocytes, and animal models have demonstrated that biofilms produced by *S. dysgalactiae* exhibit high structural stability, favoring persistent and recurrent infections, particularly in bovine mastitis [193]. The ability to form biofilms is also directly related to lower susceptibility to antimicrobial agents. The extracellular matrix hinders the diffusion of antibiotics and reduces the metabolic activity of bacteria located in the deeper layers of the biofilm, favoring the survival of bacterial subpopulations [193,194].

Another determining aspect of the evolution of *S. dysgalactiae* is the acquisition of genes through horizontal gene transfer. The presence of bacteriophages, prophagic regions, conjugative elements, and other mobile genetic elements facilitates the incorporation of genes involved in virulence and antimicrobial resistance [195]. Among these are genes encoding superantigens, DNases, and other proteins that contribute to adaptation to new hosts and increased invasive capacity [195,196,197]. The diversity of the accessory genome observed among different strains demonstrates that horizontal gene transfer is one of the main drivers of the evolution of this species.

In addition to extracellular colonization, *S. dysgalactiae* has the ability to adhere to, invade, and temporarily survive within human epithelial cells, including keratinocytes [198]. This intracellular location partially protects the bacterium from the action of antibiotics and the effector mechanisms of the immune system, contributing to the persistence and recurrence of infections [188].

### 4.5. Streptococcus uberis

Infection with *S. uberis*, a major environmental pathogen responsible for many cases of bovine mastitis, begins when the bacteria penetrate through the teat canal and adhere to the mammary gland tissues [199]. The animal’s susceptibility to this colonization peaks during the “dry period,” a phase of gland involution in which bactericidal activity and the immune response of macrophages are significantly reduced, allowing local secretions to provide ideal nutrients for bacterial proliferation. After entry and fixation in the tissue, the microorganism multiplies and triggers a strong inflammatory response, characterized by the influx of polymorphonuclear leukocytes and neutrophils into the secretory alveoli [200].

To survive and invade the host, *S. uberis* relies on a wide range of virulence factors. One of the most critical mechanisms involves the secretion of PauA (plasminogen activator), a plasminogen activator that converts bovine plasminogen into plasmin. This enzyme actively degrades milk casein, releasing peptides and amino acids essential for bacterial nutrition during the embryonic stages of colonization [201,202]. The collection of these nutrients is managed by the Opp protein transport system, while the *mtuA* gene encodes a lipoprotein vital for the sequestration of manganese, an ion fundamental for the microorganism’s growth in milk [203,204]. To protect itself against phagocytosis and intracellular destruction by neutrophils, *S. uberis* can produce a hyaluronic acid capsule, encoded by the genes *hasA*, *hasB*, and *hasC* [205]. Although most strains do not exhibit an obvious mucoid capsule, they are still pathogenic, also resorting to other tools such as the hyaluronidase enzyme and the production of the CAMP factor to enhance their virulence [111,206].

The primary step for both invasive colonization and biofilm formation is the adhesion of the bacteria to the bovine epithelium [207]. *S. uberis* uses surface receptors to bind to components of the host’s extracellular matrix, such as collagen and fibronectin. The SUAM adhesin (*Streptococcus uberis* Adhesion Molecule), a molecule of approximately 112 kDa with a very high affinity for lactoferrin, plays a key role in this process [199,208]. Lactoferrin, present in milk and tissues, acts as a “bridge” between the SUAM protein and the receptors on the epithelial cells of the mammary gland, thus facilitating firm attachment and subsequent internalization of the bacteria [209].

Once anchored, the bacteria can aggregate and begin producing extracellular polymeric substances to build biofilms, a survival strategy that makes them extremely resistant to antibiotic treatments and elimination by the host’s defense cells, largely explaining refractory and chronic mastitis [210]. In vitro assays have revealed that the mere presence of bovine milk in the medium exponentially increases the capacity for the formation of these biofilms. It was also found that the native bacterial flora present in raw milk acts synergistically, allowing *S. uberis* to develop even thicker structures [211]. The success of this bacterial community is also dictated by the nutritional environment: supplementation with carbohydrates such as fructose, glucose, and sucrose boosts biofilm production, while the presence of high levels of lactose considerably reduces it [211,212].

### 4.6. Streptococcus equi subsp. equi

Colonization by *S. equi* subsp. *equi* occurs primarily in the upper respiratory tract of equines, including horses, ponies, donkeys, and mules, which constitute the natural hosts of the bacteria [213]. Although the infection is considered highly specific to equines, occasional cases have been described in other animal species, including dogs, cats, and humans, generally associated with close contact with infected equines. These events are considered rare and accidental [213,214,215]. After entering the host, the bacterium adheres to the respiratory epithelium and extracellular matrix components through various surface proteins. Among these, the M-like protein SeM and other cell wall-anchored proteins containing the LPXTG motif stand out, promoting binding to fibronectin, collagen, and other extracellular matrix molecules, facilitating persistent colonization of respiratory tissues [216,217,218]. The collagen-binding protein (CNE) mediates binding to collagen in connective tissues, while the equi-albumin-binding protein (EAG) interacts with host albumin and immunoglobulins, contributing to adhesion and immune evasion [219,220].

Biofilm formation is another important mechanism for the persistence of *S. equi* subsp. *equi*. Bacterial organization into biofilms protects microorganisms from the action of neutrophils, antibodies, and antimicrobial agents, favoring prolonged survival in guttural pouches and explaining the chronic carrier state observed in some animals after clinical resolution of the disease [220,221]. The production of extracellular matrix rich in polysaccharides also allows for nutrient retention and the establishment of microenvironments favorable to bacterial growth [221].

Evasion of host immune defenses depends on multiple virulence factors. The hyaluronic acid capsule constitutes one of the main anti-phagocytic mechanisms, as it mimics components of the mammalian extracellular matrix, reducing immune recognition and hindering phagocytosis by neutrophils [222,223]. In association with the capsule, the SeM protein plays a central role in resistance to elimination by the immune system. This protein binds to fibrinogen and complement factor H, reducing C3b deposition on the bacterial surface and inhibiting opsonization and phagocytosis [217]. Additionally, the secreted protein Se18.9, also a factor H binder, reinforces the inhibition of complement activation and decreases the bactericidal activity of equine neutrophils [224]. Other evasion mechanisms include the protease IdeE, an enzyme capable of degrading equine immunoglobulin G (IgG), compromising the specific humoral response and reducing the effectiveness of antibody-mediated opsonization [225,226].

The intense inflammatory response characteristic of strangles largely results from the action of superantigenic exotoxins. The *S. equi* subsp. *equi* genome contains several genes encoding streptococcal superantigens, including *seeH*, *seeI*, *seeL*, and *seeM* (homologous to the *spe* genes present in other streptococcal species) [219,227,228,229]. These toxins promote polyclonal activation of large populations of T lymphocytes through simultaneous binding to the major histocompatibility complex class II and TCR receptors, triggering a massive release of pro-inflammatory cytokines [227,228,230]. Consequently, intense neutrophil recruitment, tissue necrosis, and the formation of purulent abscesses characteristic of the mandibular and retropharyngeal lymph nodes occur [227].

Thus, the pathogenesis of *S. equi* subsp. *equi* results from the coordinated action of factors involved in the adhesion and colonization of respiratory mucosa, biofilm formation, evasion of the immune response, production of inflammatory toxins, and invasion of host tissues, allowing bacterial persistence and the development of clinical manifestations characteristic of strangles.

**Figure 2:**
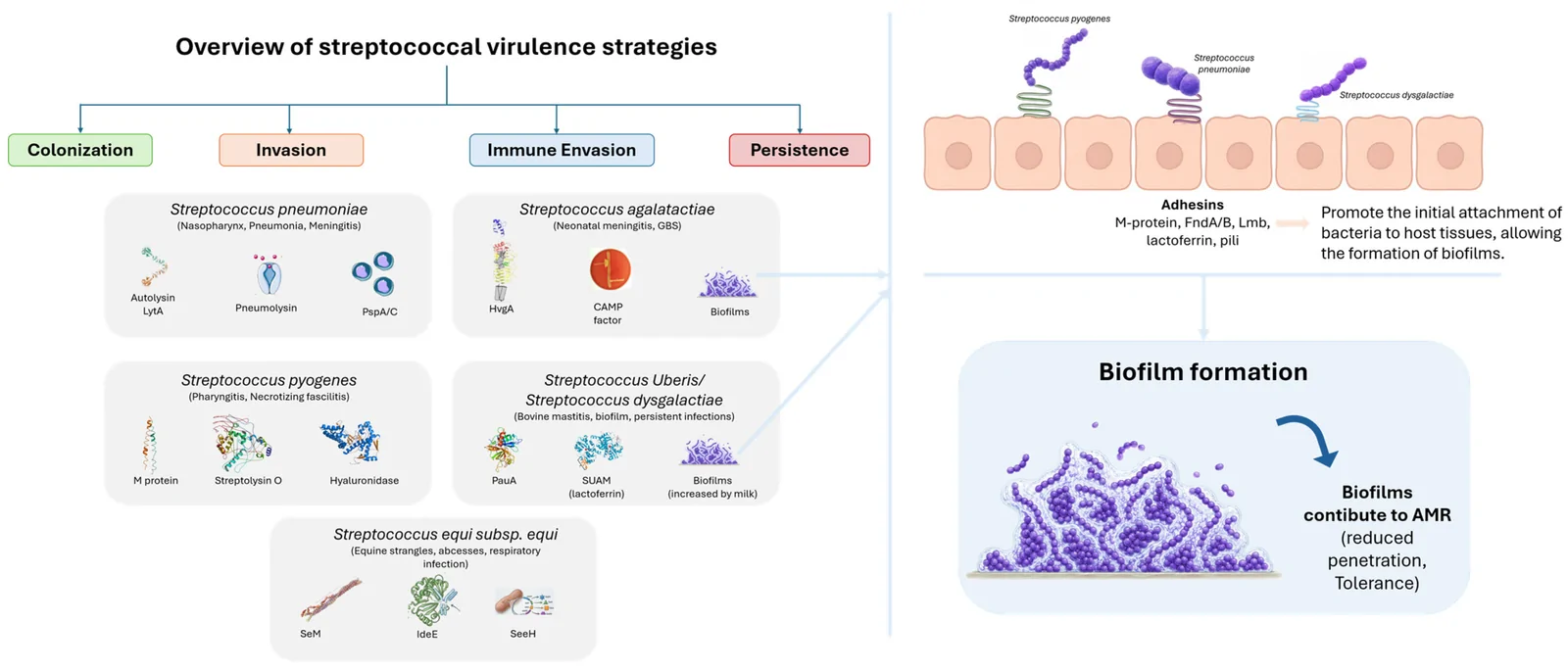
Overview of streptococcal virulence strategies. This detailed infographic organizes the virulence strategies of various *Streptococcus* species into four main categories: colonization, invasion, immune evasion, and persistence. The left side provides specific examples of virulence factors and associated diseases for human and animal streptococcal pathogens such as *S. pneumoniae*, *S. pyogenes*, *S. agalactiae*, *S. uberis*, *S. dysgalactiae*, and *S. equi* subsp. *equi*. The right side details key pathogenicity mechanisms, including adhesin-mediated adhesion to epithelial cells and biofilm formation, which is highlighted for its contribution to antimicrobial resistance through reduced agent penetration and bacterial tolerance.

The pathogenic capacity of different *Streptococcus* species is based on the expression of a diverse arsenal of specific proteins and virulence factors, whose coordinated action dictates the success of the infection. As diagrammed in Figure 2, these bacterial strategies unfold in crucial mechanistic steps: initial tissue colonization, subsequent invasion, sophisticated evasion of the host’s immune defenses, and finally, persistence in the body.

## 5. Future Prospects

Controlling infections caused by species of the genus *Streptococcus* continues to represent a significant challenge, particularly due to increasing antimicrobial resistance, the high genetic diversity of some species, and their ability to adapt to different hosts. In this context, future strategies should prioritize not only the development of new therapeutic approaches but also the prevention of infection through vaccination, genomic surveillance, and the effective implementation of the One Health concept.

Vaccine development is one of the most promising areas. Currently, only *Streptococcus pneumoniae* has widely used vaccines in human clinical practice, namely pneumococcal conjugate vaccines, which have substantially reduced the incidence of invasive disease [231]. However, the phenomenon of serotype replacement and the emergence of strains not included in vaccine formulations highlight the need to develop serotype-independent vaccines targeting conserved proteins, such as pneumolysin (Ply), pneumococcal surface protein A (PspA), and adhesion protein PsaA, allowing for more comprehensive and lasting protection [232].

Regarding *Streptococcus pyogenes*, there is still no approved vaccine for human use, despite more than a century of research. The main obstacle lies in the high variability of the M protein, one of the main virulence factors, as well as the possibility of some antigens inducing autoimmune reactions associated with rheumatic fever [233]. Currently, several vaccines based on conserved regions of the M protein and on antigens unrelated to this protein are under development, seeking to obtain transversal protection against multiple M types and minimize the risk of autoimmunity. Despite recent advances, challenges remain regarding the identification of protective correlates, standardization of clinical trials, and demonstration of efficacy in highly endemic populations [233,234].

In the case of *Streptococcus agalactiae*, the development of maternal vaccines represents one of the most promising preventive strategies. Conjugated and conserved protein-based vaccine candidates are in advanced stages of clinical development and could significantly reduce early and late neonatal disease, as well as decrease the need for intrapartum antibiotic prophylaxis, simultaneously contributing to reducing the selective pressure exerted by antibiotics [235,236].

In veterinary medicine, the situation remains different. Although commercial vaccines against *Streptococcus equi* exist, used in the prevention of equine strangles, their efficacy is variable, and safer immunogens capable of conferring longer-lasting immunity are still needed [214,227,237]. In contrast, for agents such as *Streptococcus uberis*, one of the main etiological agents of bovine mastitis, universally effective vaccines do not yet exist, despite encouraging results obtained with experimental vaccines targeting adhesion proteins, virulence factors, and components involved in biofilm formation. The limited availability of vaccines for species of veterinary importance highlights the need to intensify research in this area, since preventing infection could significantly reduce the use of antimicrobials in animal production [214,237].

Alongside vaccine development, future therapeutic approaches should focus on alternative strategies to conventional antibiotics. Among these, anti-virulence drugs stand out, capable of inhibiting factors responsible for adhesion, invasion, and biofilm formation without exerting strong selective pressure for the development of resistance. The use of bacteriophages, phage-derived endolysins, antimicrobial peptides, nanoparticles, and CRISPR-Cas-based technologies also constitutes a rapidly expanding research area, particularly relevant for the treatment of multidrug-resistant strains [238,239,240,241].

Another priority will be the integration of WGS into epidemiological surveillance programs. This technology allows for the identification of resistance genes, virulence factors, mobile genetic elements, and transmission chains with high resolution, facilitating the early detection of outbreaks and the monitoring of the spread of emerging clones among humans, animals, and the environment [242,243]. The combination of genomics with other omics approaches, such as transcriptomics, proteomics, and metabolomics, will also allow for a better understanding of host-pathogen interactions and the identification of new therapeutic and vaccine targets [244,245,246].

Finally, it is evident that the control of *Streptococcus* spp. infections will increasingly depend on an integrated One Health approach. Coordinated surveillance between human and veterinary medicine, the prudent use of antimicrobials, the strengthening of vaccination programs, and the implementation of global genomic surveillance systems will be fundamental to limiting the spread of resistant strains and reducing the impact of these bacteria on public and animal health [247,248]. In this context, multidisciplinary collaboration between researchers, clinicians, veterinarians, and health authorities will be crucial for developing sustainable strategies for the prevention and control of streptococcal infections.

## 6. Conclusions

*Streptococcus* species continue to represent a major challenge for both human and veterinary medicine due to their remarkable diversity, broad pathogenic potential, and increasing antimicrobial resistance. Although β-lactam antibiotics remain highly effective against several clinically relevant species, the widespread emergence of resistance to macrolides, lincosamides, tetracyclines, and other antimicrobial classes has progressively narrowed therapeutic options, particularly in patients requiring alternative treatments. The dissemination of mobile genetic elements, horizontal gene transfer, and the global expansion of successful resistant clones have further accelerated the evolution and spread of antimicrobial resistance across streptococcal populations.

Beyond antimicrobial resistance, the pathogenic success of *Streptococcus* spp. is largely determined by a complex repertoire of virulence factors, including adhesins, polysaccharide capsules, toxins, extracellular enzymes, and biofilm-forming capacity. The close interplay between virulence and resistance enhances bacterial persistence, facilitates immune evasion, and contributes to treatment failure, emphasizing that these characteristics should not be considered independently. Understanding the molecular mechanisms underlying both processes is essential for improving diagnostics, guiding antimicrobial therapy, and supporting the development of novel therapeutic and preventive strategies.

The increasing availability of whole-genome sequencing and other molecular surveillance tools is transforming our ability to monitor the emergence of resistant and hypervirulent lineages, identify transmission pathways, and support evidence-based public health interventions. However, continuous phenotypic surveillance remains indispensable for clinical decision-making and for validating genomic predictions.

Given the close interconnection between human, animal, and environmental reservoirs, antimicrobial resistance in *Streptococcus* spp. should be addressed within a One Health framework. Strengthening antimicrobial stewardship, expanding integrated surveillance networks, promoting vaccination when available, and fostering multidisciplinary collaboration will be critical to limiting the spread of resistant strains and reducing the burden of streptococcal infections. Future research should focus on elucidating the molecular links between antimicrobial resistance and virulence, identifying novel therapeutic targets, and developing innovative strategies capable of overcoming the growing challenge posed by these adaptable and clinically significant pathogens.

## Figures and Tables

**Table 1:** Differential diagnosis of common *streptococci*.

Bacterium	Hemolysis	Lancefield Group	Key Biochemical Test
*S. pyogenes*	β	A	Bacitracin Susceptible/PYR+
*S. agalactiae*	β	B	CAMP Test Positive
*S. pneumoniae*	α	N/A	Optochin Susceptible/Bile Soluble
*S. gallolyticus*	γ	D	Bile-Esculin Positive/No growth in 6.5% NaCl
*Viridans* Group	α/γ	N/A	Optochin Resistant/Bile Insoluble

N/A: not applicable.

**Table 2:** Reported resistance rates to the main antimicrobial classes among clinically relevant *Streptococcus* species.

Species	Antimicrobial Class					
	Penicillin (%)	Macrolides (%)	Clindamycin (%)	Tetracyclines (%)	Levofloxacin (%)	Aminoglycosides (%)
*Streptococcus pneumoniae*	17.3	19	-	20	3–5	<1
*Streptococcus pyogenes* (GAS)	0	15–30	15–30	20–30	1–5	<1
*Streptococcus agalactiae* (GBS)	1.7	30–40	25–35	80	50	<1
*Viridans* group *streptococci* (VGS)	10–15	20–50	20–50	60–80	5–10	<1
*Streptococcus dysgalactiae* (Groups C/G)	0	10–20	10–20	20–30	2–5	<1

## Data Availability

No new data were created or analyzed in this study. Data sharing is not applicable to this article.
